# CAR-T Cells in the Treatment of Urologic Neoplasms: Present and Future

**DOI:** 10.3389/fonc.2022.915171

**Published:** 2022-07-04

**Authors:** Zhengchao Zhang, Dong Li, Heng Yun, Wei Liu, Keqiang Chai, Jie Tong, Tongwei Zeng, Zhenghua Gao, Yongqiang Xie

**Affiliations:** ^1^ Department of Urology, The Third Affiliated Hospital of Gansu University of Traditional Chinese Medicine, Baiyin, China; ^2^ Department of General Surgery, Second Hospital of Lanzhou University, Lanzhou, China

**Keywords:** CAR-T cells, urologic neoplasms, kidney cancer, prostate cancer, bladder cancer, immunotherapy

## Abstract

In recent years, with the breakthrough of CAR-T cells in the treatment of hematological tumors, they are increasingly being used to treat solid tumors, including urologic neoplasms. There are many relatively specific targets for urologic neoplasms, especially prostate cancer. Besides, urologic neoplasms tend to progress more slowly than tumors in other organs of the body, providing ample time for CAR-T cell application. Therefore, CAR-T cells technology has inherent advantages in urologic neoplasms. CAR-T cells in the treatment of urologic neoplasms have been extensively studied and preliminary achievements have been made. However, no breakthrough has been made due to the problems of targeting extra-tumor cytotoxicity and poor anti-tumor activity. we systematacially summarized the research actuality of CAR-T cells in urologic neoplasms, discussed the potential value and difficulties of the research. The application of CAR-T cells in the treatment of urologic neoplasms requires improvement of function through screening for better targets, modification of CAR structures, or in combination with other antitumor approaches.

## Introduction

Urologic neoplasms mainly include prostate cancer, bladder cancer, kidney cancer, adrenal tumor, penile tumor, testicular tumor and ureter tumor. According to GLOBOCAN in 2018, the incidence and mortality of urologic neoplasms were 12.3% and 7.7%, respectively (1). At present, prostate cancer, bladder cancer and kidney cancer are the most common urinary system tumors. The early stage of tumor occurrence can be successfully treated by surgery or radiotherapy and chemotherapy, but there is no radical treatment for advanced tumor (2–4). In the past decade, immunotherapy has emerged as a new direction in the treatment of advanced urologic neoplasms (5–7). Since urologic neoplasms are usually slow-growing compared to the other systemic tumors, there is a time window for treatment, which provides conditions for the selection of appropriate immunotherapy. In addition, tumor-associated antigens such as prostate stem cell antigen (PSCA), prostate specific membrane antigen (PSMA) and epidermal growth factor receptor (EGFR) are expressed in urologic neoplasms. Therefore, the choice of CAR-T cells for urologic neoplasms has an innate advantage.

CAR-T cells technology is a superior immunotherapy for cancer that delivers antitumor effects by providing genetically modified T cells that precisely target tumor-associated antigens (8). CAR-T cells represent a major advance in the field of tumor immunotherapy with their success in treating B-cell-derived lymphoma and leucocythemia (9). Since then, CAR-T cells have become increasingly popular in the treatment of solid tumors. Since then, the study of CAR-T cells in solid tumor therapy has gradually become a hotspot. However, there is considerable heterogeneity between solid and hematologic tumors. First, it is difficult to find tumor-specific targets in solid tumors such as CD19 of hematological tumor cells, and only tumor-associated antigens can be applied, which might led to the emergence of CAR-T cells targeting extratumoral cytotoxicity. Secondly, the drug has a good diffusion effect in the blood system, and it is easy for the drug to contact the tumor cells. Nevertheless, solid tumors have a dense stromal component and an immunosuppressive microenvironment. It is difficult for CAR-T cells to fully infiltrate tumor tissue and contact with tumor cells. Even if CAR-T cells are infiltrated into tumor tissue, and they have to overcome the problem of immunosuppression to exert tumor killing function (10). To overcome these challenges, structural optimization and target screening of CAR-T cells are under investigation.

Urologic neoplasms, like other solid tumors, have been widely used in immunotherapy in recent years, and some progress has been made. Although CAR-T cells offer an innovative approach for the treatment of patients with advanced urinary tumors, no product has been successfully introduced into the clinic. A large number of researchers have attempted to overcome this dilemma by optimizing target selection, modifying CAR-T structure, improving tumor immune microenvironment, and combining other molecular targeted therapies. This review will systematically introduce the research progress and potential value of CAR-T cells in urinary system tumors, and discuss the difficulties faced, in order to provide new ideas for the treatment of urinary system tumors.

## CAR-T Cells Technology

**Figure 1:**
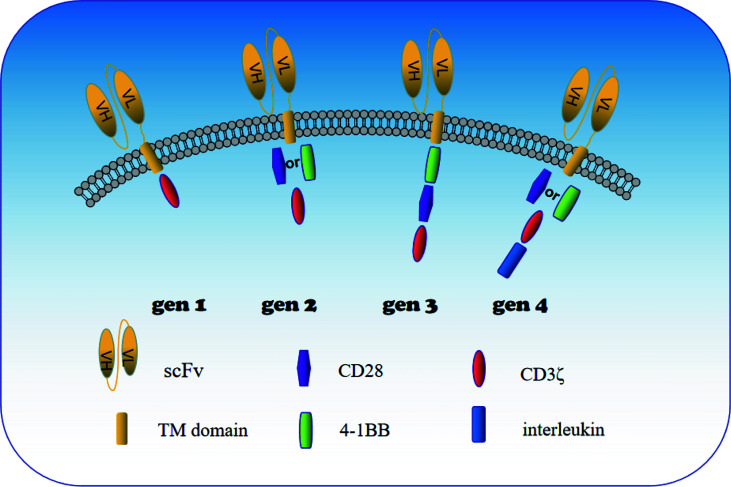
Schematic diagram of the construction of 1st to 4th generation CAR-T cells. When CAR-T cells enter tumor tissue during tumor therapy, SCFV fragments specifically bind to homologous antigens on the surface of tumor cells. The activation signal then passes through the transmembrane domain to the intracellular costimulatory domain, and then activates T cells by activating the costimulatory molecule to finally kill the tumor cells. SCFV fragment (extracellular domain), CD8 (transmembrane domain) and CD3ζ (intracellular domain) are the basic structures of CAR T cells. Generation-to-generation CAR-T cells are optimized for T cell proliferation and tumor killing by increasing intracellular costimulatory molecules. The CD3ζ costimulation domain was the only intracellular domain of the first generation CAR-T cells. The second generation of CAR-T cells significantly enhanced their cytotoxicity by adding a costimulatory molecule (CD28 or 4-1BB) over the first generation. The intracellular domain of third-generation CAR-T cells contains two costimulatory molecules in order to enhance activation of T cells. The fourth generation CAR-T cells have increased intracellular co-expressed cytokines in addition to the second generation CAR-T cells, and co-expressed cytokines mainly positively regulate the proliferation and differentiation of CAR T cells and recruit peripheral immune cells to better kill tumor cells.

CAR-T cells are regarded as the representative of adoptive immunotherapy. The greatest advantage is that they can bind directly to the surface of cancer cells without major histocompatibility complex (MHC) restriction and induce tumor cell death (11). In recent years, CAR-T cells have exhibited impressive therapeutic effect in the treatment of B cell malignancies (12). As a result, researchers have focused on CAR-T cells technology in the application of solid tumors, including urologic neoplasms. CAR-T cells consist of three major components: an extracellular domain (SCFV fragment) that recognizes an antigen of tumor, a transmembrane domain (CD8), and an intracellular domain (costimulatory molecules) that mediates activation of T lymphocytes (13) (**Figure 1**). When CAR-T cells enter tumor tissue during tumor therapy, SCFV fragments specifically bind to homologous antigens on the surface of tumor cells. The activation signal then passes through the transmembrane domain to the intracellular costimulatory domain, and then activates T cells by activating the costimulatory molecule to finally kill the tumor cells. Generation-to-generation CAR-T cells are optimized for T cell proliferation and tumor killing by increasing intracellular costimulatory molecules. The CD3ζ costimulation domain was the only intracellular domain of the first generation CAR-T cells. The second generation of CAR-T cells significantly enhanced activation of T cells by adding a costimulatory molecule (CD28 or 4-1BB) over the first generation (14). It was found that CD28 activated T cells had a strong instantaneous mortality, while 4-1BB activated T cells had better anti-tumor persistence (15).The intracellular portion of third-generation CAR-T cells contains two costimulatory molecules(CD28 and 4-1BB) in order to enhance activation of T cells. With the development of genetic engineering technology, the intracellular structure of CAR-T cells changed dramatically, and four generations of CAR-T cells were generated according to the structure (**Figure 1**).The fourth generation CAR-T cells have increased intracellular co-expressed cytokines (IL-7, IL-18, IL-21, CCL19, PH40, etc) base on the second generation CAR-T cells, aiming to positively regulate CAR-T cells (16). Generation 2 and 4 CAR-T cells have been widely studied in solid tumors. 2 generation CAR-T cells showed stable function and easy manipulation. 4 generation CAR-T cells not only regulate the immunosuppressive microenvironment of solid tumors, but also directly participate in positive regulation of T cell activation and proliferation (17–19). Therefore, 4-generation CAR-T cells are the key development direction of solid tumors in the future.

T cells used for CAR-T cell therapy are usually taken from the patient’s peripheral blood (20),and were first separated from the obtained peripheral blood and then infected with lentiviruses carrying CAR plasmids. Finally, CAR-T cells were expanded *in vitro* and reinjected into patients. The production process of CAR-T cells also raises many questions for clinical use. On the one hand, due to organ function exhaustion in patients with advanced tumor, peripheral blood immune cell activity will be inhibited, which will affect the activity of CAR-T cells produced. In addition, the economic cost of CAR-T cell customization is expensive, which is difficult to bear for the majority of patient population. In order to reduce the cost of producing CAR-T cells and increase their convenience, researches are also underway on universal CAR-T cells, which will make this technology available to a wider patient population (21).

## CAR-T Cells for Urologic Neoplasms

Ten years ago, tumor immunotherapy has been widely used in preclinical research and treatment of urinary system tumors, including cytokines and tumor vaccines. Since then, immune checkpoint inhibitors have also shown some efficacy in the treatment of urologic neoplasms. However, a large number of clinical studies have found that these treatments do not significantly improve patient prognosis. In recent years, with the breakthrough of CAR-T cells in the treatment of hematological tumors (13, 22), CAR-T cells technology has been gradually applied in the research of solid tumors, including urologic neoplasms. CAR-T cells also provides a new approach for the treatment of urologic neoplasms such as kidney cancer, prostate cancer, bladder cancer and so on. Many preclinical and clinical trials have been conducted for CAR-T cells in urologic neoplasms (**Tables 1**, **2**), and a portion of clinical trials are ongoingin in the hope of achieving satisfactory results.

**Table 1:** Preclinical studies of CAR-T cells in urologic neoplasms.

Tumour	Targeted antigen	Structure of CAR	Reference
Renal Carcinoma	CAIX	fourth–generation (PD-L1 antibodies)	Eloah Rabello Suarez et al, 2016 (23)
Renal Carcinoma	CAIX	second –generation(4-1BB)	Huizhong Li et al, 2020 (24)
Renal Carcinoma	c-MET	third –generation(CD28/4-1BB)	Jun-ich Mori et al, 2021 (25)
Prostate Carcinoma	PSMA	second –generation(4-1BB)	Christopher C. Kloss et al, 2018 (26)
Prostate Carcinoma	PSCA	second –generation(4-1BB)	Saul J. Priceman et al, 2018 (27)
Prostate Carcinoma	PSMA	second –generation(CD28)	Jamal Alzubi et al, 2020 (28)
Prostate Carcinoma	PSMA	fourth –generation (IL-23)	Dawei Wang et al, 2020 (29)
Prostate Carcinoma	B7-H3	second –generation(CD28)	Yida Zhang et al, 2021 (30)
Bladder Cancer	PD1	second –generation(CD28)	Geoffrey Parriott et al, 2020 (31)
Bladder Cancer	MUC1	second –generation(CD28)	Lei Yu et al, 2021 (32)
Bladder Cancer	EGFR	second –generation(CD28)	Camilla M. Grunewald et al, 2021 (33)

**Table 2:** Clinical trials of CAR-T cells in urologic neoplasms.

Conditions	Targeted antigen	Phase	Number Enrolled (n)	NCT Number	Locations	Status
Renal Carcinoma	AXL/ROR2	I/II	66	NCT03393936	Shanghai Public Health Clinical Center , Shanghai, China	Active, not recruiting
Renal Carcinoma	VEGFR2	I/II	24	NCT01218867	National Institutes of Health Clinical Center, Maryland, United States	Terminated
Renal Carcinoma	c -MET	I/II	73	NCT03638206	The First Affiliated Hospital of Zhengzhou University Zhengzhou, Henan, China	Recruiting
Prostate Carcinoma	PSCA	I	33	NCT03873805	City of Hope Medical Center Duarte, California, United States	Recruiting
Prostate Carcinoma	PSMA	I	40	NCT04249947	City of Hope Comprehensive Cancer Center Duarte, California, United States	Recruiting
Prostate Carcinoma	PSMA	I	18	NCT04768608	The First Affiliated Hospital, Zhejiang University Hangzhou, Zhejiang, China	Not yet recruiting
Prostate Carcinoma	PSMA	I	50	NCT04227275	Moffitt Cancer Center Tampa, Florida, United States	Active, not recruiting
Prostate Carcinoma	PSCA	I/II	151	NCT02744287	Moffitt Cancer Center Tampa, Florida, United States	Recruiting
Prostate Carcinoma	EpCAM	I/II	60	NCT03013712	IEC of Chengdu Medical College Chendu, China	Unknown
Prostate Carcinoma	NKG2DL	I	10	NCT04107142	Landmark Medical Centre Johor Bahru, Johor, Malaysia	Not yet recruiting
Prostate Carcinoma	PSMA	I	18	NCT03089203	University of Pennsylvania Philadelphia, Pennsylvania, United States	Active, not recruiting
Bladder Cancer	PSMA	I/II	20	NCT03185468	Shenzhen Second People Hospital Shenzhen, Gongdong, China	Recruiting
Bladder Cancer	ROR2	I	18	NCT03960060	Shanghai Zhongshan Hospital Shanghai, Shanghai, China	Active, not recruiting
Bladder Cancer	HER2	I	45	NCT03740256	Baylor St. Luke’s Medical Center Houston, Texas, United States	Recruiting
Bladder Cancer	HER2	I	18	NCT04660929	Abramson Cancer Center Philadelphia, Pennsylvania, United States	Recruiting

All clinicaltrials were download at www.clinicaltrials.gov (access date: April 3, 2022).

As shown in **Table 1**, targets selected of preclinical studies for CAR-T cells in the treatment of urologic neoplasms mainly include CAIX, PSMA, PSCA, and other generic targets of solid tumors. The structure of CAR is mainly the second generation, and the selected costimulatory molecules are mainly CD28 and 4-1BB. Relatively few third- generation and fourth-generation CAR-T cells have been used to treat urologic neoplasms.

### Renal Cell Carcinoma

Worldwide, renal cell carcinoma (RCC) is the 9th most common malignancy in men and the 14th most common malignancy in women. After more than 20 years of increasing incidence, the incidence of RCC worldwide has shown signs of stabilizing or even declining in recent years. However, in the United States, the incidence of RCC continues to increase, mainly for early-stage tumors, and the overall mortality rate for RCC is stabilizing (34). For decades, the only effective treatment for RCC was surgery, as RCC was largely resistant to cytotoxic chemotherapy and insensitive to radiation, which makes the search for new antitumor therapies a priority. Based on the in-depth research on tumor immunity, immunotherapy has become a promising alternative method (35, 36).

Cytokines (IL-2 and IFN-α) as nonspecific immunotherapy have long been the standard treatment for metastatic renal cell carcinoma (mRCC). However, most studies of IL-2 and IFN-α as adjuvant immunotherapy in recent years have shown negative results (37–39). Furthermore, the immune checkpoint inhibitors (ICIs) has shown more favorable efficacy and safety in RCC than second-line chemotherapy (40). Over the past decade, the treatment of patients with mRCC has changed substantially, with pre-combination therapies based on immunotherapy replacing targeted therapies. However, the study also found the importance of ICIs in combination with other anti-tumor therapies. A network meta-analysis conducted by Fahad Quhal et al. (41) showed that the combination of ICIs and tyrosine kinase inhibitors (TKIs) provided better PFS, ORR and OS than the ICIs alone. More recently, data from the ICIs combined anti-tumor trial also confirmed the survival benefit of ICIs combined with pembrolizumab in the treatment of mRCC. These combination therapies are recommended as first-line treatment for advanced renal cancer by the Updated 2021 Guidelines of the European Association of Urology (42). Immunotherapy in combination with other therapies has been approved for the treatment of kidney cancer, and more studies are needed to evaluate their efficacy and safety to guide selection of the best first-line treatment.

The achievement of CAR-T cells in hematologic oncology has prompted the application of CAR-T cells in RCC (43). To date, numerous studies have been conducted on the association of CAR-T cells in the treatment of RCC. Eloah Rabello Suarez et al. (23) exploited a targeted carbonic anhydrase IX (CAIX) CAR-T cells. In a humanized mouse model of mRCC, tumor growth and mass were significantly reduced after treatment with CAX-CAR-T cells. Furthermore, Jun-Ich Mori et al. (25) constructed c-Met-targeted CAR-T cells and validated the antitumor efficacy of c-Met-CAR-T cells *in situ* mouse models derived from clinical renal papillary cell carcinoma tissues. The c-Met-CAR-T cells have been demonstrated to infiltrate tumor tissues and inhibit tumor growth. CD70 has also been found to be highly expressed in RCC and limited in normal tissue, making it an attractive target for CAR-T immunogenic solid tumors (44). Recently, Siler H Panowski et al. (45) constructed CAR-T cells targeting CD70 single-chain antibodies. CD70-CAR- T cells showed strong antitumor activity against RCC cell lines and patient-derived xenograft mouse models. These studies indicate the potential value of CD70-CAR-T cells in treating RCC, and a phase I clinical trial of CD70-CAR-T cells in treating metastatic renal cell carcinoma is ongoing.

CAR-T cells in combination with other anti-tumor methods are also a focus of research. To enhance CAR-T cells antitumor activity, Huizhong Li et al. (24) combined CAX-CAR-T cells and sunitinib showed significant synergistic effect in the mRCC mouse xenotransplantation model. The combination group exhibited greater proliferation and tumor killing than mice treated alone. This combination approach may provide meaningful insights into CAR-T cell therapy for urologic neoplasms. Jun-Ich Mori et al. (25) also evaluated the anti-RCC efficacy of c-MET-CAR-T cells in combination with axitinib, and found that axitinib synergically enhanced the anti-tumor efficacy of CAR-T cells. It suggests that CAR-T cells combined with targeted drugs may also be a way to treat solid tumors in the future. In addition, Chen Zhang et al. (46) studied the binding of a decorin-carrying oncolytic adenovirus(OAV-decorin) to CAIX-CAR-T to perform antitumor activity against renal cancer cells. Oav-decorin in combination with CAIX-CAR significantly reduced tumor load, altered extracellular matrix (ECM) composition by inhibiting collagen fiber distribution, reduced TGF-β expression, enhanced IFN-α secretion, and generated more CAR-T cells. The combined treatment model also prolonged the survival of the mice. These data also confirmed the role of oncolytic adenovirus and CAIX-CAR-T cells against solid tumors.

In 2016, the National Institutes of Health clinical Center in Maryland published a clinical study of VEGFR2-targeted CAR T cells in the treatment of metastatic kidney cancer(NCT01218867). A total of 24 patients were enrolled, of whom 5 (20.83%) had severe adverse reactions and 1 (4.17%) died. Five years of follow-up showed that 1 patient had partial response, 1 patient had stable disease, and the rest had tumor progression. In this study, researchers also divided the patients into groups according to different doses of combined IL-2. The 5 patients with severe complications were all in the high-dose IL-2 group, while no serious complications were found in the low-dose IL-2 group. The efficacy of VEGFR2-CAR-T cells in mRCC was not satisfactory. This study demonstrates that VEGFR2-CAR T cells are not satisfactory in the treatment of mRCC, but the side effects are acceptable.

Nevertheless, Lamers et al. (47) implemented a phase I/II trial of targeting CAIX CAR-T cells(first generation) to investigate the safety and efficacy of these cells in the treatment of mRCC. Unfortunately, due to the expression of the target antigen in intrahepatic bile duct epithelium, resulting in targeted out-of-tumor cytotoxicity, some patients have discontinued treatment due to detection of liver damage. This study suggests that the selection of tumor-associated antigens for CAR-T cells therapy in solid tumors is particularly important, and that normal tissue cytotoxicity to the selected target antigens must not cause major organ damage in patients. It also indicates that there is still a long way to go for CAR-T cells therapy in solid tumors, especially in the process of step-by-step target selection.

Fortunately, there are a number of clinical studies underway. There are currently two ongoing CAR-T cells studies (NCT03393936, NCT03638206) in China for the treatment of metastatic renal cancer (**Table 2**), with AXL,ROR2 and c-MET as the target, respectively. The objective was to assess the safety and efficacy of CAR-T cells in the treatment of mRCC.

### Prostate Carcinoma

Worldwide, prostate cancer (PCa) is the second most common cancer in men and the fifth deadliest cancer in men (48). Over the past 20 years, great advances in surgery, radiotherapy and hormone therapy for PCa have significantly reduced mortality from the disease. However, metastatic castration resistant prostate cancer (mCRPC) is still very difficult to cure. Although docetaxel, abiraterone acetate, and radiotherapy have been shown to enhance patient outcomes in combination with standard hormone therapy, studies have shown that this subset of patients is rarely cured and has severe side effects (49, 50).

It is urgent to develop new therapeutic modalities for mCRPC patients. Given the success of immunotherapy in the treatment of many malignant tumors in recent years, immunotherapy of mCRPC is being widely explored. Over the past decade, researchers have made great efforts to explore this therapeutic area. Tumor vaccines have been widely used in preclinical and clinical studies of mCRPC, mainly including DC vaccines (51, 52), viral vector vaccines (53, 54) and DNA/mRNA vaccines (55, 56). Clinical studies have shown that some vaccines alone can prolong overall survival, while others require a combination of other therapies to slow tumor progression. In addition, ICIs are widely used in the treatment of mCRPC. In mCRPC patients, pembrolizumab was found to have antitumor activity and a reasonable safety profile as a standard monotherapy. However, Graff et al. (57) found that the effective rate of anti-PD-1 treatment for mCRPC was less than 30%. Therefore, the use of ICIs in mCRPC is still limited by their low clinical immune response rate (58–60). Since then, researchers have begun to investigate the efficacy of CAR-T cells for mCRPC and hope that CAR-T cell therapy will lead to a breakthrough in the treatment of mCRPC.

There are many tumor specific antigens in PCa tissues, such as prostate specific antigen (PSA), PSMA and PSCA. PSMA is a type II transmembrane glycoprotein expressed in the membrane of prostatic epithelium. PSMA was highly expressed in prostate tissue and solid tumor blood vessels, but hardly expressed in normal tissues such as intestine, liver and kidney (61). PSCA is a tumor-associated antigen found in PCa cells (62). PSCA is expressed on the membrane of the prostate gland and can only be detected there. The expression rate of PSCA in PCa tissue was much higher than that in normal prostate tissue (63). In addition, PSCA expression was not detected in other normal tissues (64). PSCA has also become an important target for targeted therapy of PCa. These studies suggest that PCa is a favorable tumor for CAR-T cells therapy (65).

Many studies have been conducted in recent years due to the inherent advantages of CAR-T cells technology in PCa. In 2008, researchers constructed targeting PSMA first generation CAR-T cells first time in a clinical trial (NCT00664196) of five patients. Only two patients achieved a clinical partial response, with PSA reductions of 50% and 70%, respectively. No toxicity against PSMA was observed. It was also found that low plasma doses of IL2 did not support antitumor activity under optimal CAR T cell implantation (66). The efficacy of the first generation CAR-T cells was found to be poor, mainly due to poor persistence of CAR-T cells.

To further optimize the efficacy of CAR-T cells, Qiangzhong Ma et al. constructed second generation PSMA-CAR-T cells containing CD28 stimulating molecule, it showed stronger anti-tumor response in mouse models (67). In a related clinical study (NCT01140373), 2 of 4 patients were stable and 2 patients were advanced. The results of this study showed that the second-generation CAR-T cells were well tolerated and significantly improved the efficacy. In addition, to select better costimulatory molecules, Saul J. Priceman et al. (27) constructed targeting PSCA CAR-T cells, using different costimulatory molecules (CD28 and 4-1BB), and compared the sensitivity of the two intracellular costimulatory molecules to tumor antigen expression. PSCA-CAR-T cells exhibit potent *in vivo* antitumor activity. Compared with CAR-T cells containing CD28, CAR-T cells containing 4-1BB showed better T cell persistence and disease control because they expressed higher tumor antigen intensity. These result show that CAR-T cells targeting PSMA and PSCA are well tolerated in the treatment of PCa and may have good efficacy. These studies suggest that CAR-T cells targeting PSMA and PSCA, accompanied by structural optimization, may have good efficacy in the treatment of PCa.

More recently, Vivek Narayan et al. (68) reported the results of a phase I clinical trial (NCT03089203) of PSMA CAR-T cells against castrated PCa. Five of the 13 patients developed grade 2 cytokine release syndrome, and three other patients achieved a 30% PSA reduction. Therefore, the study also showed that clinical use of targeted PSMA-CAR-T cells is feasible and generally safe. However, the antitumor activity of CAR-T cells in PCa still needs to be enhanced. Dawei Wang et al. (29) constructed IL23-PSMA-CAR-T cells. IL23-PSMA-CAR-T cells exhibited significantly more proliferation and cytokine secretion *in vitro* and also exhibited faster tumor clearance and weight gain *in vivo* than conventional CAR-T cells. CAR-T cells that co-express cytokines are a potential approach to enhance their antitumor activity in the treatment of PCa.

Furthermore, CAR-T cells were used in combination with chemotherapy to enhance tumor killing activity. Jamal Alzubi et al. (28) designed of CAR-T cells targeting PSMA. *In vivo*, local injection of PSMA-CAR-T cells eradicated xenograft PCa in mice. In addition, systemic intravenous CAR-T cells combined with low-dose docetaxel chemotherapy significantly inhibited tumor growth, whereas docetaxel alone or CAR-T cells did not. Studies have demonstrated that the combination of PSMA-CAR-T cells with chemotherapy is a promising immunotherapy pathway for the clinical treatment of mCRPC.

Radiation therapy is an vital treatment for PCa, and PCa stem cells (PCSCs) have the ability to resist radiation. Recently, Yida Zhang et al. (30) found that radiotherapy up-regulated the expression of PCSCs and immune checkpoint B7-H3 in each PCa cell line. They constructed CAR -T cells targeting B7-H3 and validated their antitumor activity *in vivo* and *in vitro*. The results exhibited that B7-H3-CAR-T cells were more cytotoxic to PCSCs than PCa cells. In immunodeficient mice, radiotherapy combined with B7-H3-CAR-T cells was more effective than radiotherapy or CAR-T cells alone. This study demonstrates the importance of using CAR-T technology to target antigens produced or increased during tumor therapy. This suggests that CAR-T cells technology has great potential in combination with other antitumor technologies.

There are a number of ongoing trials involving CAR-T cells for PCa (**Table 2**), both in China and the United States. The selection of targets focused on PSMA, PSCA, EpCAM and NKG2DL. The objective of the clinical study was to evaluate the safety and efficacy of different gene-editing CAR-T cell technologies in the treatment of mCRPC. Most clinical studies are in the process of being recruited and are expected to be effective.

### Bladder Cancer

Bladder cancer (BC) is the 10th most common cancer worldwide. BC has about 430,000 new cases diagnosed each year (69). Smoking and sex are known risk factors for BC (70). According to the clinical TNM classification of malignant tumors, there are three types of BC: non-muscle-invasive BC (NMIBC), muscle-invasive BC (MIBC) and metastatic BC(MBC) (70). More than 70% of the new BC patients were diagnosed with NMIBC, with the remainder diagnosed with MIBC or MBC. Generally speaking, the 5-year survival rate for NMIBC is as high as nearly 90%, but the 5-year survival rate for MIBC drops sharply to no more than 50% or even less than 50%, and less than 15% for MBC (71, 72). Therefore, different types of urothelial carcinoma are treated differently. NMIBC can be treated by tranurethral bladder tumor resection, after which intravesical BCG or adjuvant chemotherapy can be selected (73). The preferred regimens for MIBC are radical resection and cisplatin based neoadjuvant chemotherapy (74). Intravenous chemotherapy is considered the best treatment option for MBC patients. Although surgery, radiation, chemotherapy, and targeted therapies have made some progress in the treatment of BC over the past 30 years, the prognosis for MBC patients remains poor (75).

Immunotherapy has been used to treat BC for the last 10 years. Such as BCG vaccine (76) and ICIs (77, 78). Intravesical BCG therapy is now standard practice for NMIBC, including carcinoma *in situ* and high-grade papillary neoplasms (79). ICIs were used in BC include Atezolizumab (80, 81), Avelumab (82), Durvalumab (83, 84) Nivolumab (85)and Pembrolizumab (86). At present, these drugs are mainly used for second-line treatment when chemotherapy is ineffective. However, only about 20 percent of patients show an immune response in clinical trials. In addition, it has also been reported that ICIs can cause serious adverse events, with at least 10% of patients experiencing serious adverse events (87). As a result, only a small number of people have benefited clinically from this approach. With the rapid development of tumor immunotherapy, adoptive immunotherapy and other immunotherapy methods have been developed in the treatment of BC.

CAR-T cells as an adoptive immunotherapy approach require tumor target antigens. Tumor associated antigens are expressed on the surface of tumor cells and represent potential therapeutic targets, BC cells are rich in tumor-associated target antigens. In addition, several studies have found that PSMA expression is observably higher in BC than in healthy urothelium (88, 89). Such as HER2, MUC1, EGFR as tumor targets of pan-cancer, are also highly expressed in BC tissues and can be used as a therapeutic target of BC (90, 91). In conclusion, CAR-T cells technology for BC treatment does not lack tumor-associated antigen.

CAR-T cell technology has undergone extensive preclinical and clinical studies in the treatment of urothelial carcinoma. Geoffrey Parriott et al. (31)developed targeting PD1 CAR-T cells that recognizes PD1 receptor ligand expressed in a variety of solid cancers. The results showed that PD1-CAR-T cells lysed tumor cells and resulted in long-term tumor-free survival in mice. Recently, Lei Yu et al. (32)constructed targeting MUC1 CAR-T cells, and verified the immunotherapeutic response *in vitro* by using BC. Specific cytotoxicity occurred only in MUC1 positive organs such as BC. The success of this study verified the feasibility of using MUC1-CAR-T cells in the clinical treatment of BC.

In order to enhance the antitumor activity of CAR-T cells, some researchers also combined decitabine with EGFR-targeting CAR-T cells to conduct anti-bladder tumor studies, and the study found that the combination can enhance the tumor-specific killing of BC (33). Therefore, CAR-T cells combined with DNA methylation specific inhibitors is also a method to enhance the anti-solid tumor function, and its efficacy needs to be confirmed by further clinical studies. Understanding the determinants of CAR-T cell recognition of tumors is important to improve CAR- T cell function. Greco, B et al. (92) found that a variety of cancers expressed extracellular N-glycan, and its abundance was negatively correlated with CAR-T cell killing activity. Further studies showed that N-glycan protects tumors from CAR-T cells by interfering with appropriate immune synapse formation, reducing transcriptional activation, cytokine production, and cytotoxicity. To overcome this obstacle, researchers took advantage of the high metabolic requirements of tumors to safely inhibit N-glycan synthesis. In xenograft mouse models of pancreatic and BC, such treatment disrupted n-glycan coverage on tumor cells, leading to enhanced CAR-T cell activity. These studies indicate that exploring the mechanism of tumor regulating the response intensity of CAR-T cells is also an important direction to achieve breakthroughs in the treatment of solid tumors.

Currently, clinical studies of CAR-T cells for BC are ongoing (**Table 2**). These clinical programs are being funded in China and the United States. The target selection includes HER2, PSMA, and ROR2. The objective of the clinical study is to evaluate the safety and efficacy of gene editing car-T cells with different targets in the treatment of MBC. However, due to the small number of clinical studies on bladder cancer, few studies have published results.

## Potential Value and Dilemma of CAR-T Technology in Urologic Neoplasms

Tumor associated antigens play an vital role in the application of CAR-T cells technology in cancer therapy, and urologic neoplasms have a relatively high number of specific targets compared to other organ tumors in the body. AIX, PMSA, and PSCA-targeted CAR T cells have demonstrated tumor-killing activity in several preclinical studies, and clinical studies have demonstrated that PMSA-CAR-T cells can be tolerated in clinical use and have good antitumor activity (66).. Secondly, many studies have confirmed that tumor-associated target antigens commonly expressed in solid tumors are expressed in urologic neoplasms, including MUC1, EGFR, VEGFR2, EpCAM, C-Met, NKG2DL, MUC1, etc. Therefore, the use of CAR-T cells in urologic neoplasms has an advantage over other systemic tumors in the selection of target antigens.

The clinical application of CAR-T cell technology requires the collection of peripheral blood monocytes from patients, and the selected T cells need to be transfected with Lentiviruses carrying CAR plasmids, and finally applied to patients. This process takes a certain amount of time, and urologic neoplasms develop relatively slowly in systemic tumors, providing ample time for CAR-T cells to be used. Due to the pathogenesis characteristics of urologic neoplasms, multiple treatments can be performed with CAR-T with different targets. In addition, most preclinical studies of CAR-T cells have found that local administration is superior to intravenous administration, which may be due to limited tumor tissue infiltration and enrichment capacity of CAR-T cells. However, it is feasible to use local drugs in the treatment of urologic neoplasms.

It is well known that the progression of urologic neoplasmsis relatively slow and the prognosis is relatively good in all major systemic tumors. As a result, the development of complementary therapies for advanced tumors has been slow, and there have been relatively few studies of CAR-T in urologic neoplasms. As shown in **Table 2**, current studies mainly focus on PCa, while there are few studies on kidney cancer and bladder cancer. At present, the number of clinical studies on CAR-T in solid tumors such as digestive tumors and gliomas is larger than in urologic neoplasms. Published studies of CAR-T in the treatment of urologic neoplasms face the same problems as other solid tumors. For example, Lamers et al. (47) conducted the I/II clinical trial of CAIX-targeted CAR-T therapy for metastatic renal cancer, which was terminated due to abnormal liver function in most patients. In addition, Vivek Narayan et al. (68) reported a phase 1 trial of PMSA-CAR-T cell therapy for PCa (NCT03089203), in which 1 of 13 patients developed grade 4 CRS with sepsis and died, and only 3 patients achieved a 30% PSA reduction. Therefore, the current problems faced by CAR-T cells in the treatment of urologic neoplasms are mainly the poor anti-tumor activity and targeted extratumoral cytotoxicity. Therefore, in future studies, the selection of target antigens should be further optimized to alleviate the problem of targeting extratumoral cytotoxicity, and then the structure of CAR-T should be optimized to enhance the tumor killing activity of CAR-T on the basis of avoiding cytokine syndrome.

## Discussion

CAR-T cell therapy has been extensively studied in the urinary tumor, most of which are preclinical studies. After all, a large number of clinical studies are needed to verify the efficacy and clinical complications of CAR T cells before they can be used in the clinic. Therefore, more clinical studies of CAR-T cells in urologic neoplasms are needed in the future. Furthermore, although there are many tumor-associated targets for urological CAR-T cells, published studies have demonstrated a lack of specificity. Targeting extratumoral cytotoxicity has been a major challenge in solid tumor therapy using tumor-associated antigens to construct CAR-T cells. Tumor- associated antigens should be optimized to select targets with high expression in tumor tissues and low expression in other non-important organs, so as to effectively kill tumor cells without causing serious complications to patients.

The immunosuppressive tumor microenvironment has been identified as one of the biggest obstacles to the successful treatment of urologic neoplasms with CAR-T cells. Gene-editing of CAR-T cells with positive immunomodulator and immunosuppressor antibodies is a strategy to overcome this obstacle. Keishi Adachi et al. (17) designed gene-editing IL-7 and CCL19 CAR-T cells (IL-7/CCL19-CAR-T cells). Because these immunomodulatory factors are critical for the maintenance of T cell regions in lymphatic organs, they may be involved in the regulation of tumor immunosuppression microenvironments. IL-7/CCL19-CAR-T cells exerted superior antitumor activity *in vivo* compared to conventional CAR-T cells. Recently, Xingcheng Xiong et al. (93) also designed gene-editing IL-7 and PH20 CAR-T cells(IL-7/PH20-CAR-T cells). Coexpressed PH20 can effectively degrades the extracellular matrix and enhances the tumor-infiltrating function of T cells. Studies have shown that IL-7/PH20-CAR-T cells significantly enhance their antitumor activity in multiple solid tumors. These techniques can be used to enhance the efficacy of CAR-T cell technology against urologic neoplasms.

In addition, CAR-T cells combined with molecularly targeted drugs are a promising way to treat urologic neoplasms in the future. For example, Huizhong Li et al. (24) Combined treatment with AIX-CAR-T and Sunitinib have demonstrated synergistic efficacy in mRCC mouse xenograft model. It was found that sunitinib not only up-regulated the expression of CAIX in tumor cells, but also reduced the myeloid suppressor cells in the tumor microenvironment. Jun-Ich Mori Et al. (25) also combined c-Met-CAR-T cells with axitinib, which once again demonstrated that molecular targeted drugs can synergically enhance the antitumor efficacy of CAR-T cells. Preclinical trials using CAR-T cells in combination with chemotherapy and radiation for PCa have shown significant mutually reinforcing effects (28, 30). These findings suggest that CAR-T cells technology in combination with other antitumor technologies has great potential in the treatment of urologic neoplasms.

## Conclusions

In conclusion, numerous studies have demonstrated the potential value of CAR-T cells in urologic neoplasms. However, due to immunosuppressive microenvironment and physical barriers in tumor tissue, CAR-T cells still have poor invasion and persistence in urologic neoplasms. In addition, targeting extratumoral cytotoxicity is also an important issue in the application of CAR-T in urologic neoplasms. Therefore, relevant studies need to further optimize the selection of targets, and CAR-T cells may be more capable of killing urologic neoplasms through gene-editing cytokines, combined molecular targeting agents, and chemotherapy. It is believed that with the further study of tumor immune mechanism, CAR-T cells will achieve satisfactory results in the treatment of urinary system tumors.

## Author Contributions

All authors conceptualized and wrote the manuscript. ZZ and DL additionally performed literature and data analysis. All authors contributed to the article and approved the submitted version.

## Funding

This study was funded by the Third Affiliated Hospital of Gansu University of Chinese Medicine (Project Approval Number: 2016YG-06) and Baiyin City 2019 Science and technology plan project (Project Approval Number: 2019-1-22Y)

## Conflict of Interest

The authors declare that the research was conducted in the absence of any commercial or financial relationships that could be construed as a potential conflict of interest.

## Publisher’s Note

All claims expressed in this article are solely those of the authors and do not necessarily represent those of their affiliated organizations, or those of the publisher, the editors and the reviewers. Any product that may be evaluated in this article, or claim that may be made by its manufacturer, is not guaranteed or endorsed by the publisher.

## Abbreviations

CAR-T cells, chimeric antigen receptor T cells; PSCA, prostate stem cell antigen; PSMA, prostate specific membrane antigen; EGFR, epidermal growth factor receptor; MHC, major histocompatibility complex; RCC, renal cell carcinoma; mRCC, metastatic renal cell carcinoma; ICIs, immune checkpoint inhibitors; TKIs, tyrosine kinase inhibitors; CAIX, carbonic anhydrase IX; OAV-decorin, decorin-carrying oncolytic adenovirus; ECM, extracellular matrix; PCa, prostate cancer; mCRPC, metastatic castration resistant prostate cancer; PSA, prostate specific antigen; BC, bladder cancer; NMIBC, non-muscle-invasive bladder cancer; MIBC, muscle-invasive bladder cancer; MBC, metastatic bladder cancer
